# The Experience of Self-Diagnosing with Autism Spectrum Condition Amongst Adult Females: A Qualitative Study

**DOI:** 10.1192/bjo.2026.11212

**Published:** 2026-06-30

**Authors:** Lara Arif, Metodi Siromahov, Will Mandy

**Affiliations:** University College London, London, United Kingdom

## Abstract

**Aims:**

Autistic females are less likely to receive an autism diagnosis in childhood than autistic males. In recent years, increasing numbers of adults have come to identify as autistic prior to, or in the absence of, a formal diagnosis. While existing research has examined experiences of receiving an autism diagnosis in adulthood, comparatively little is known about how adults come to self-diagnose, the meanings they attach to this process, and how self-diagnosis shapes decisions about seeking formal assessment.

**Methods:**

This qualitative study involved 30 female participants aged 18–43 who had, at some point, identified as autistic without holding a formal diagnosis. Participants took part in one-to-one, online, semi-structured interviews exploring their understandings of autism, pathways to self-diagnosis, perceived barriers and facilitators, attitudes towards both self- and professional diagnosis, and intentions regarding formal assessment. Interview transcripts were analysed using Framework Analysis.

**Results:**

Framework Analysis identified six interrelated themes.

First, participants described self-diagnosis as *a prolonged sense-making process* involving extensive reflection and information-seeking, challenging portrayals of self-diagnosis as impulsive or trend-driven.

Second, accounts reflected *heterogeneous social representations of autism*: older, male-centred stereotypes were frequently rejected, while gender differences were instead mapped onto alternative frameworks such as high–low functioning distinctions.

Third, self-diagnosis was commonly experienced as *an “aha” moment* of epistemic and affective clarity following extended uncertainty, countering social contagion narratives.

Fourth, an *economy of legitimacy* shaped participants’ experiences. Structural barriers to professional diagnosis, including gendered underdiagnosis and lengthy waiting lists, positioned self-diagnosis as a necessary substitute for institutional validation, while media discourses framing self-diagnosis as “trendy” or as social contagion undermined its credibility and generated self-doubt.

Fifth, participants adopted *a reflexive stance towards self-diagnosis*, emphasising its provisional status, distancing themselves from “frivolous” self-identification, and expressing ambivalence about the label; social media was viewed as both informative and epistemically suspect.

Finally, *attitudes towards professional diagnosis* were ambivalent: a strong desire for validation coexisted with fears of dismissal, misrecognition, or gendered misdiagnosis, shaped by material and emotional barriers and mediated by social relationships influencing disclosure and decisions about seeking assessment.

**Conclusion:**

Self-diagnosis functions as a meaningful response to diagnostic exclusion rather than a trivial or trend-driven practice. Recognising its role has important implications for clinical practice, service access, and how autism is understood beyond traditional diagnostic pathways.

